# Determinants of undernutrition among young children in Ethiopia

**DOI:** 10.1038/s41598-022-25160-y

**Published:** 2022-12-05

**Authors:** Biniyam Sahiledengle, Lillian Mwanri, Pammla Petrucka, Abera Kumie, Girma Beressa, Daniel Atlaw, Yohannes Tekalegn, Demisu Zenbaba, Fikreab Desta, Zinash Teferu, Debebe Wordofa, Kenbon Seyoum, Degefa Gomora, Getahun Negash, Kingsley Emwinyore Agho

**Affiliations:** 1 Department of Public Health, Madda Walabu University Goba Referral Hospital, Bale-Goba, Ethiopia; 2 Centre for Public Health Research, Equity and Human Flourishing, Torrens University Australia, Adelaide Campus, Adelaide, SA 5000 Australia; 3 College of Nursing, University of Saskatchewan, Saskatoon, Canada; 4 School of Public Health, College of Health Science, Addis Ababa University, Addis Ababa, Ethiopia; 5 Department of Human Anatomy, Madda Walabu University Goba Referral Hospital, Bale-Goba, Ethiopia; 6 Department of Midwifery, Madda Walabu University Goba Referral Hospital, Bale-Goba, Ethiopia; 7 Department of Medical Laboratory Science, Madda Walabu University Goba Referral Hospital, Bale-Goba, Ethiopia; 8 School of Health Sciences, Western Sydney University, Locked Bag 1797, Penrith, NSW 2751 Australia; 9 Translational Health Research Institute, School of Medicine, Western Sydney University, Campbelltown Campus, Penrith, NSW 2571 Australia; 10 African Vision Research Institute, University of KwaZulu-Natal, Durban, 4041 South Africa

**Keywords:** Diseases, Health care, Risk factors

## Abstract

Ethiopia is one of the countries in sub-Saharan Africa with the highest burden of childhood undernutrition. Despite the high burden of this scourge, little is known about the magnitude and contributing determinants to anthropometric failure among children aged 0–23 months, a period regarded as the best window of opportunity for interventions against undernutrition. This study examined factors associated with undernutrition (stunting, wasting, and underweight) among Ethiopian children aged 0–23 months. This study used a total weighted sample of 2146 children aged 0–23 months from the 2019 Ethiopian Mini Demographic and Health Survey. The data were cleaned and weighted using STATA version 14.0. Height-for-age (HFA), weight-for-height (WFH), and weight-for-age (WFA) z-scores <  − 2 SD were calculated and classified as stunted, wasting, and underweight, respectively. Multilevel mixed-effects logistic regression models adjusted for cluster and survey weights were used. Adjusted odds ratio (AOR) and 95% confidence interval (CI) were estimated. Statistical significance was declared at *p* < 0.05. The overall weighted prevalence of stunting, wasting, and underweight respectively were 27.21% [95% CI (25.32–29.18)], 7.80% [95% CI (6.71–9.03)], and 16.44% [95% CI (14.90–18.09)] among children aged 0–23 months in Ethiopia. Female children were less likely to be associated with stunting [AOR: 0.68, 95% CI (0.54–0.86)], wasting [AOR: 0.70, 95% CI (0.51, 0.98)], and underweight [AOR: 0.64, 95% CI (0.49, 0.83)] than their male counterparts. Conversely, older children aged 12–17 months [AOR: 2.22, 95% CI (1.52, 3.23)] and 18–23 months [AOR: 4.16, 95% CI (2.75, 6.27)] were significantly at an increased odds of becoming stunted. Similarly, the likelihood of being underweight was higher in older age groups: 6–11 months [AOR: 1.74, 95% CI (1.15, 2.63)], 12–17 months [AOR: 2.13, 95% CI (1.40, 3.24)], and 18–23 months [AOR: 4.08, 95% CI (2.58, 6.44)] compared with the children younger than 6 months. Lower wealth quintile was one of the other significant determinants of stunting and underweight. The study’s findings indicated that the most consistent significant risk factors for undernutrition among children aged 0–23 months are: male sex, older age groups and lower wealth quintile. These findings emphasize the importance of strengthening nutrition-specific and sensitive interventions that address the immediate and underlying drivers of childhood undernutrition in early life, as well as targeting low-income households with male children, in order for Ethiopia to meet the Sustainable Development Goals (SDGs) 1,2 and 3 by 2030.

## Introduction

Undernutrition, which includes stunting, wasting, and underweight is a major public health concern, especially in many low and middle-income countries^1^. Low height-for-age is known as stunting, and it can be caused by poor nutrition in utero and/or early childhood. Wasting refers to a condition where a child is thin for their height. A child who is underweight (low weight-for-age), may be stunted, wasted, or both^2,3^. Any form of undernutrition in children aged 0 to 23 months is a serious threat because its consequences including physical, psychological and socioeconomic impacts can last into adulthood and are irreversible^4^.

Globally in 2020, about 149 million children under the age of five were stunted, and 45 million were estimated to be wasted^3^. Notably, Sub-Saharan Africa is home to the majority of children who are undernourished in the world, and the east African region where Ethiopia is located has been reported to have the highest prevalence of undernutrition^5–7^.

The first 1000 days of a child's life (0–23 months) are a critical period of rapid physical and mental development. The damage caused by malnutrition during the first 1,000 days has a negative impact on the child's health, cognitive development, physical growth, and school and work performance later in life^8^. A closer look of existing evidence reveal that children aged 0 to 23 months have been reported to be disproportionately affected by various types of malnutrition^4,9–12^.

In Ethiopia, malnutrition contributes more than 50 percent of all infant and child deaths^8^, and according to the recent 2019 Ethiopian mini Demographic and Health Survey (EMDHS), the prevalence of stunting (37%), wasting (7%) and underweight (21%) was unacceptably high^13^. Moreover, existing primary studies in different parts of the country have reported high burden of undernutrition in children aged 0–23 months- with prevalence of stunting (21.8%-42⋅7%)^11,14–16^, wasting (9.9%-11.6%)^16^, and underweight (27.7%)^16^. Ethiopia as a nation is still dealing with a number of underlying vulnerabilities for childhood malnutrition, including food insecurity^17,18^, caused by multifactorial determinants such as prolonged drought and famine, poverty and civil unrest^19–21^.

According to the 2018 Global Nutrition Report, country progress toward global targets can be tracked using determinants such as child wasting, stunting, and exclusive breastfeeding, making undernutrition a significant indicator of a nation’s socioeconomic developmental status. Ethiopia is one of 100 countries on track to achieve none of these targets^22^. In Ethiopia, undernutrition remains a major concern, and the country suffers from a substantial burden of acute and chronic malnutrition in early childhood^23–25^. In order to address undernutrition, the Ethiopian government is implementing several initiatives to combat childhood malnutrition including the National Nutritional Plans I and II and the Seqota Declaration. For instance, the recent Seqota Declaration has made statement to end stunting in Ethiopia for children under two years old through effective coordination and collaboration of sectors, leading to the broader vision "to end stunting in children under two years in Ethiopia by 2030^25^.

Undernutrition has been reported to be associated with multiple factors: sex of the child^26^, age of the child^26–28^, birth size^29,30^, birth order^28^, short birth interval^31^, lack of exclusive breastfeeding^32^, having repeated diarrheal episodes^32^, lack of improved sanitation facilities^31,33^, maternal body mass index (BMI)^27,31,32^, maternal education^29,30^, and wealth status^29,30,34^ are among the factors associated with childhood stunting. Similarly, different studies have elicited the predictors of wasting in children, such as sex of the child^27,35^, birth size^36^, cough^27^, fever^37^, maternal BMI^36^, maternal education^27,38^, diarrheal morbidity^35,38,39^, complementary food starting before 6 months^38^, and unimproved drinking water^40^. Furthermore, sex of the child^41,42^, child age^42,43^, diarrheal morbidity in the last two weeks^41–43^, paternal education^41,43^, preceding birth interval^43^, birth order^42^, type of birth^42^, maternal body mass index^42^, birth size of child^42^, duration of breastfeeding^42^, and wealth index^42,43^ had a significant effect on underweight.

Although childhood malnutrition in Ethiopia has been widely reported, evidence on the relative strength of its determinants focusing on early in life (0–23 months) has not been adequately investigated, as previous studies have mainly examined the problem among children 0–59 months generally^30,37,40,42,44,45^. Also, early studies have put more emphasis on socioeconomic disparity^46^, stunting^29,30,37^, wasting^40^, underweight^42^, or spatial variations^31,40^.

Previous studies have concentrated on specific types of undernutrition in children aged 0–56 or 6–59 months^29–31,37,40,47^. Furthermore, existing evidence in Ethiopia that shows problem of malnutrition during the first 0–23 months was limited to local areas^10,16,48–50^, with some focusing on anaemia^51,52^, and levels of dietary diversity^53^. As far as our knowledge, there have been no studies conducted exclusively among children aged 0 to 23 months, using nationally representative data and rigorous analysis. Therefore, this study aimed to assess the determinants of undernutrition (stunting, wasting, and underweight) among children aged 0–23 months in Ethiopia. Identifying the determinants in this “critical window of opportunity” period is important for interventions against undernutrition and to meet the Sustainable Development Goals (SDGs) by 2030, in particular SDG 2, which aims to end hunger, achieve food security and improve nutrition.

## Methods

### Study design and setting

The current study used the 2019 Ethiopia Mini Demographic and Health Survey (EMDHS). The EMDHS) used a cross-sectional design and is the latest survey addressing childhood health issues in Ethiopia. Ethiopia is located in the horn of Africa. Its geographical coordinates are 9.145° N latitude and 40.4897° East longitude. The country covers an area of 1.1 million square Kilometers. Administratively, Ethiopia is divided into eleven geographical regions and two administrative cities (namely, Addis Ababa, Afar, Amhara, Benishangul-Gumuz, Dire Dawa, Gambella, Harari, Oromia, Somali, Southern Nations and Nationalities and People [SNNP], Tigray, Sidama, and South West Ethiopia Peoples' Region). In this analysis, Sidama and South West Ethiopia Peoples' regions were under South Nations and Nationalities Peoples region. Oromia, Amhara, and SNNP are highly populous states that account for 37·9%, 21·6%, and 21·3% of the country’s population, respectively^54^.

### Sampling and population

The 2019 Ethiopia Mini Demographic and Health Survey (EMDHS) is the second Mini Demographic and Health Survey conducted in Ethiopia. Data collection took place from 21 March 2019 to 28 June 2019^13^. The data was based on the nationally representative sample that provided estimates at the national and regional levels and for urban and rural areas. The sampling frame used for the 2019 EMDHS is a frame of all census enumeration areas (EAs) created for the 2019 Ethiopia Population and Housing Census (EPHC). The census frame is a complete list of the 149,093 EAs created for the 2019 EPHC. An EA is a geographic area covering an average of 131 households. The 2019 EMDHS sample was stratified and selected in two stages. In the first stage, a total of 305 EAs (93 in urban areas and 212 in rural areas) were selected proportionally, considering the EA size. In the second stage of selection, a fixed number of 30 households per cluster were selected with an equal probability systematic selection from the newly created household listing. In all selected households, height and weight measurements were collected from children age 0–59 months^13^. The 2019 EMDHS gathered anthropometric data from children under five (n = 5,279 for stunting, n = 5,408 for wasting, and n = 5,338 for underweight)^13^. For the analysis of the current study, a total of 2,146 children aged 0–23 months who had valid and complete anthropometric measurements were included.

### Data collection

In the anthropometry questionnaire, height and weight measurements were recorded for eligible children aged 0–59 months in all interviewed households. Weight measurements were obtained using lightweight, electronic SECA 874 scales with a digital screen and the mother and child function. Height measurements were performed using measuring boards. Children younger than age 24 months were measured lying down (recumbent) on the board. Health professionals were trained to measure children’s height and weight. Training on child height measurement included standardization exercises^13^.

### Variables and measurement

Stunting, wasting, and underweight were taken separately as dependent variables with binary categories. All anthropometric failure outcomes were constructed based on the 2006 World Health Organization (WHO) child growth standards^1^. Stunting was defined as a height-for-age z score less than − 2 standard deviations (SDs) of the median, wasting as a weight-for-height z score less than − 2 SDs, and underweight as a weight-for-age z score of less than − 2 SDs^1^.

Individual, household and community-level factors were considered as the potential determinants variables. The individual-level factors included were child sex, child age, number of under-five children, birth order, birth interval, dietary diversity score, meal frequency, breastfeeding status, received vitamin A in last 6 months, maternal age (years), maternal education, antenatal care, place of delivery. Household-level factors include household wealth, household size, household toilet facility, and household’s source of drinking water. Community-level factors include place of residence and region.

Minimum dietary diversity is a proxy for adequate micronutrient density of foods. Minimum dietary diversity defined as the proportion of children age 6–23 months who received a minimum of five out of eight food groups during the previous day. The five groups should come from a list of eight food groups: breast milk; grains, roots, and tubers; legumes and nuts; dairy products (milk, yogurt, and cheese); flesh foods (meat, fish, poultry, and liver/organ meat); eggs; vitamin A-rich fruits and vegetables; and other fruits and vegetables. Minimum meal frequency was defined as proportion of children age 6–23 months who received solid, semisolid, or soft food (including milk feeds for non-breastfed children) the minimum number of times or more during the previous day.

Minimum meal frequency is a proxy for meeting energy requirements. Breastfed children aged 6–8 months are considered to be fed with a minimum meal frequency if they receive solid, semisolid, or soft foods at least twice a day. Breastfed children aged 6–23 months are considered to be fed with a minimum meal frequency if they receive solid, semisolid, or soft foods at least three times a day. Non-breastfed children aged 6–23 months are considered to be fed with a minimum meal frequency if they receive solid, semisolid, or soft foods or milk feeds at least four times a day and if at least one of the feeds is a solid, semisolid, or soft food^55^.

Toilet facility was categorized as “Improved", "Unimproved" or "Open defecation. Facilities would be considered improved if any of the following occurred: flush/pour flush toilets to piped sewer systems, septic tanks, and pit latrines; ventilated improved pit (VIP) latrines; pit latrines with slabs; and composting toilets. Unimproved facilities included: flush or pour-flush to elsewhere; pit latrine without a slab or open pit; bucket, hanging toilet or hanging latrine. Other facilities, including households with no facility or use of bush/field, were considered as open defecation^56^. Source of drinking water was categorized as “Improved”, or “Unimproved”. Improved sources of drinking water included piped water, public taps, standpipes, tube wells, boreholes, protected dug wells and springs, and rainwater. Other sources of drinking water were regarded as unimproved^56^. The principal components statistical procedure was used to determine the weights for the wealth index based on the number and kinds of consumer goods they own, ranging from a television to a bicycle or car, and housing characteristics such as source of drinking water, toilet facilities, and flooring materials. This index was divided into quintiles categories, and the bottom 40% of household wealth index factor score were classified as the poorest households, the next 20% as the middle-class households and the top 40% as the rich households, as used in the past studies^6^.

### Data extraction and analysis for the current study

#### Data extraction

The EMDHS, 2019 household member recode (PR) file, a nationally representative large-scale dataset, served as the data source for this analysis. To gain access to the EMDHS-2019 dataset, we used the study title and significance to download the data from the Measure DHS website at www.measuredhs.com after receiving permission for registration. This was followed by the extraction of a wide range of information about potential individual and community level factors.

### Data analysis

Analyses were performed using STATA version 14.0 Statistical software (StataCorp. 2015. Stata Statistical Software: Release 14. College Station, TX: StataCorp LP). Given the complexity of the two-stage sampling design of EMDHS, sample weighting was used to account for stratification and clustering for precision. For the complex sample design, it is necessary to consider three types of information: (i) the primary sampling unit or cluster variable, (ii) the stratification variable, and (iii) the weight variable to ensure that the estimates are representative at the national level. Data cleaning was performed prior to the analysis to ensure that our findings were consistent with the number of young children categorized in the final Ethiopia Mini-DHS 2019 report and according to the three anthropometric indices of nutritional status: height-for-age, weight-for-height, and weight-for-age. Descriptive analysis was conducted to describe the prevalence of undernutrition according to the independent variables. Multilevel mixed effect models were particularly suitable for our analysis where data for participants were organized at more than one level due to the hierarchical nature of DHS data (i.e., nested data). With such cluster data, children within a cluster may be more similar to each other than children in the rest of the cluster. As a result, we applied a multilevel logistic regression model to assess the association between determinants and undernutrition (stunting, wasting, and underweight). All possible covariates with a *p*-value of less than 0.25 in the multilevel bi-variable analysis were included in the multilevel multivariable logistic regression models. To avoid or minimize statistical error, multicollinearity was tested. In this study, the variance inflation factor was less than 10, so there was no multicollinearity collinearity between independent variables.

For the analysis, four models were used. The primary model (empty or null model) was fitted without explanatory variables. The second model (individual level factors), third model (community-level factors), and fourth model (final or full model) were adjusted for individual and community-level factors simultaneously. Adjusted odds ratio (AOR), along with their corresponding 95% confidence intervals (CIs), were used to estimate the strength and direction of the association between the determinants and outcome variables (stunting, wasting, and underweight). Statistical significance was defined as a *p‐*value of less than 0.05.

For model comparison, we used the log-likelihood ratio (LL) and the Deviance Information Criterion (DIC). The DIC was used as a measure of how well our different models fitted the data with a lower value on DIC indicating a better fit of the model. Moreover, Akaike Information Criterion (AIC) and the Bayesian Information Criterion (BIC) were used as diagnostics to determine the goodness of fit. After the values for each model of AIC and BIC were compared, the lowest one was thought to be a better explanatory model. Intra-cluster correlation coefficient (ICC), median odds ratio (MOR), and proportional change in variance (PCV) statistics were calculated to measure the variation between clusters^57,58^. ICC is the measure of variation attributed to contextual neighborhood factors (such as residential level factors) and is often used to operationalize the concept of contextual phenomena^59^. MOR is used to measure unexplained cluster heterogeneity and it is the median value of the odds ratio between the clusters at low and high risk of outcome variables. PCV is used to measure the total variation of each outcome explained by the final model^57,59^.

### Ethical considerations

The data were downloaded and analyzed after the purpose of the analysis was communicated and approved by MEASURE DHS. After the approval, we downloaded this study data from http://www.dhsprogram.com. Ethics approval and participants consent were not required, as this is publicly available data.

### Ethics approval and consent to participate

The data were obtained via online registration to measure the DHS program and downloaded after the purpose of the analysis was communicated and approved. An approval letter for the use of the EDHS data set was gained from MEASURE DHS. All methods were carried out in accordance with relevant guidelines and regulations.

## Results

### Socio demographic characteristics of the study's participants

A total of 2,146 (weighted sample) children aged 0–23 months were included in this study. Almost half (50.5%) of these children were males. One in four sample children were infants (6–11 months old). Most of the children (82.1%) had a preceding birth interval of 24 months and above. The majority of children were from rural areas (73.1%). Of all the study participants, 86.0% were currently breastfeeding. About 21.5% of the children were from the poorest households while 21.2% were from the richest households. The overall prevalence of minimum meal frequency and minimum dietary diversity practice was 46.8% and 10.2%, respectively (Table 1).

**Table 1:** Distribution of stunting, wasting, and underweight by categories of selected variables among children aged 0–23 months in Ethiopia, EMDHS 2019.

Characteristics	Total Weight Freq. N (%)	Prevalence of Stunting, n (%)	Prevalence of Wasting, n (%)	Prevalence of Underweight, n (%)
**Child-related factors**				
**Age (months)**				
< 6	561 (26.1)	89 (16.0)	48 (30.2)	52 (15.3)
6–11	517 (24.1)	132 (23.6)	31 (19.4)	86 (25.3)
12–17	583 (27.2)	170 (30.5)	49 (30.5)	119 (34.9)
18–23	484 (22.6)	167 (29.9)	31 (19.8)	84 (24.6)
**Sex**				
Male	1,021 (50.5)	316 (58.3)	101 (64.4)	204 (61.4)
Female	1,001 (49.5)	226 (41.7)	56 (35.6)	128 (38.6)
**Number of under-five children**				
1	806 (39.9)	195 (36.1)	46 (29.8)	118 (35.5)
2+	1,216 (60.1)	346 (63.9)	110 (70.2)	214 (64.5)
**Birth order**				
1	468 (23.1)	123 (22.8)	14 (8.9)	63 (19.1)
2	421 (20.8)	110 (20.4)	32 (20.9)	65 (29.7)
3	285 (14.1)	77 (14.3)	22 (14.4)	42 (12.7)
4	230 (11.4)	59 (10.8)	16 (10.4)	38 (11.5)
5+	616 (30.5)	171 (31.6)	71 (45.3)	128 (36.9)
**Birth interval (n = 1,513)**				
< 24 months	277 (17.9)	84 (21.4)	25 (17.5)	53 (19.7)
≥ 24 months	1,270 (82.1)	327 (79.6)	118 (82.5)	215 (80.3)
**Vitamin A in last 6 months (n = 1,991)**				
Yes	1,324 (66.5)	340 (63.7)	122 (79.3)	219 (67.1)
No	667 (33.5)	194 (36.3)	32 (20.6)	107 (32.9)
**Currently breastfeeding**				
Yes	1,739 (86.0)	457 (84.4)	130 (83.3)	272 (81.8)
No	283 (14.0)	84 (15.6)	26 (16.7)	60 (18.2)
**Minimum meal frequency**				
Yes	1,006 (46.8)	285 (51.1)	51 (41.5)	154 (45.0)
No	1,141 (53.2)	273 (48.9)	110 (68.5)	188 (55.0)
**Minimum dietary diversity**				
Yes	205 (10.2)	53 (9.8)	7 (4.4)	28 (8.4)
No	1,817 (89.8)	489 (90.2)	150 (95.6)	304 (91.6)
**Maternal factors**				
**Age (years)**				
< 20	159 (7.9)	54 (10.1)	8 (5.1)	40 (12.1)
20–34	1,486 (73.5)	400 (73.9)	111 (71.1)	216 (65.1)
35–49	377 (18.6)	86 (16.0)	37 (23.8)	76 (22.8)
**Educational level**				
No education	937 (46.3)	286 (52.9)	93 (59.6)	189 (56.8)
Primary	805 (39.8)	212 (39.3)	51 (32.5)	130 (39.1)
Secondary	179 (8.8)	28 (5.2)	10 (6.5)	9 (2.7)
Higher	101 (5.0)	14 (2.6)	2 (1.3)	5 (1.4)
**ANC visit**				
No ANC	499 (25.3)	137 (26.2)	53 (35.8)	100 (31.2)
1–3	598 (30.3)	149 (28.5)	53 (35.9)	111 (34.7)
4+	877 (44.4)	237 (45.3)	42 (28.3)	109 (34.1)
**Place of delivery**				
Home	897 (44.9)	266 (49.3)	89 (57.3)	184 (55.4)
Health facility	1,099 (55.1)	274 (50.1)	67 (42.6)	148 (44.6)
**Household-level factors**				
**Household size**				
1–4	675 (33.4)	168 (31.0)	32 (20.8)	103 (31.1)
5+	1,347 (66.6)	373 (69.0)	124 (79.2)	229 (68.9)
**Wealth index**				
Poorest	435 (21.5)	121 (22.3)	50 (32.2)	95 (28.6)
Poorer	434 (21.5)	125 (23.0)	37 (23.9)	76 (20.9)
Middle	378 (18.7)	125 (23.1)	16 (10.4)	63 (18.9)
Richer	344 (17.0)	99 (18.3)	29 (19.0)	58 (17.5)
Richest	429 (21.2)	72 (13.2)	22 (14.4)	40 (12.1)
**Toilet facility**				
Improved	337 (17.0)	63 (12.0)	23 (14.5)	53 (16.1)
Unimproved	1,048 (52.8)	193 (36.4)	68 (44.1)	148 (44.9)
Open defecation	600 (30.2)	273 (51.6)	65 (41.4)	128 (38.9)
**Source of drinking water**				
Improved	1,282 (64.1)	351 (65.8)	108 (69.5)	207 (62.9)
Surface water	314 (15.7)	69 (12.9)	26 (16.8)	63 (19.1)
Unimproved	405 (20.2)	114 (21.3)	21 (13.7)	59 (18.0)
**Community-level factors**				
**Residence**				
Urban	543 (26.9)	105 (19.6)	37 (23.8)	82 (24.9)
Rural	1,478 (73.1)	436 (80.4)	119 (76.2)	249 (75.1)
**Region**				
Tigray	149 (7.4)	46 (8.5)	20 (12.8)	39 (11.8)
Afar	31 (1.5)	9 (1.7)	5 (2.9)	7 (2.0)
Amhara	414 (20.5)	152 (28.0)	37 (23.7)	110 (33.3)
Oromia	786 (38.9)	189 (34.9)	41 (26.3)	78 (23.6)
Somali	124 (6.2)	20 (3.7)	20 (13.1)	21 (6.4)
Benishangul	23 (1.2)	7 (1.3)	1 (1.1)	5 (1.6)
SNNP	404 (20.0)	107 (19.8)	28 (17.9)	65 (19.5)
Gambela	9 (0.4)	1 (0.2)	2 (1.1)	1.8 (0.5)
Harari	5 (0.3)	1 (0.2)	1 (0.1)	1 (0.2)
Addis Ababa	61 (3.0)	6 (1.1)	1 (0.3)	2 (0.5)
Dire Dawa	12 (0.6)	2 (0.3)	1 (0.4)	1 (0.4)

### Prevalence of stunting

**Figure 1:**
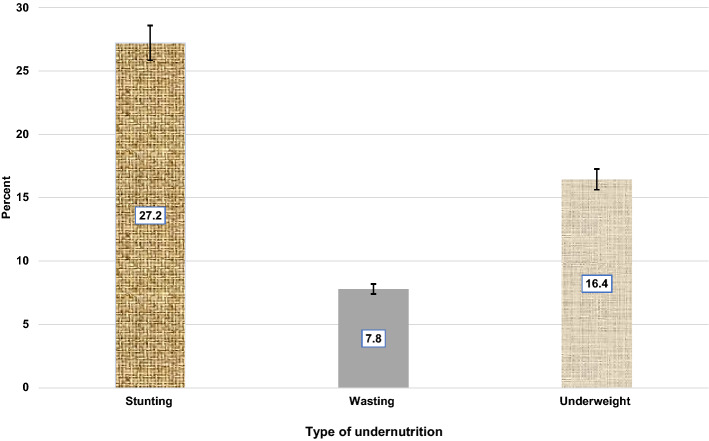
Undernutrition among children 0–23 months of age in Ethiopia, EMDHS-2019.

The prevalence of stunting was found to be 27.21% [95% CI (25.32–29.18)]. The prevalence of stunting generally differed by sex with 58.3% of stunted children were males. The proportion of stunted children was higher among uneducated mothers (52.9%) and rural residents (80.4%). Children in rural areas were more likely to be stunted compared to those in urban areas (80.4% versus 19.6%) (Fig. 1, Table 1).

### Prevalence of wasting

The prevalence of wasting was found to be 7.80% [95% CI (6.71–9.03)]. Almost two-thirds (64.4%) of wasted children were males and 76.2% lived in rural areas. Moreover, 68.5% and 95.6% of children that lacked minimum meal frequency and dietary diversity were wasted, respectively (Fig. 1, Table 1).

### Prevalence of underweight

The prevalence of underweight was found to be 16.44% [95% CI (14.90–18.09)]. About 61.4% of underweight children were males, 34.9% were between the age of 12–17 months, and 75.1% of underweight children lived in rural areas (Fig. 1, Table 1).

### Factors associated with stunting, wasting, and underweight

The result of the multilevel bivariable logistic regression analysis for stunting, wasting, and underweight is shown in Table 2 and Supplementary File 1.

**Table 2:** Bivariable multilevel logistic regression analysis of factors associated with childhood stunting, wasting, and underweight among children aged 0–23 months in Ethiopia, EMDHS 2019.

Characteristics	Stunted			Wasting			Underweight		
	Proportion, 95% CI	Unadjusted OR (95% CI)	*p*-value	Proportion, 95% CI	Unadjusted OR (95% CI)	*p*-value	Proportion, 95% CI	Unadjusted OR (95% CI)	*p*-value
**Child-related factors**									
**Age (months)**									
< 6	17.2 (14.3–20.6)	Ref		30.9 (24.9–37.5)	Ref		18.1 (14.5–22.3)	Ref	
6–11	20.8 (17.7–24.4)	1.46 (1.06–2.02)	0.021	22.2 (17.0–28.4)	0.70 (0.46–1.08)	0.111	22.8 (18.9–27.4)	1.41 (0.97–2.03)	0.069
12–17	30.6 (26.9–34.5)	2.18 (1.60–2.97)	*p* < 0.001	26.1 (20.5–32.5)	0.77 (0.51–1.16)	0.223	28.9 (24.6–33.8)	1.68 (1.18–2.39)	0.004
18–23	31.3 (27.6–35.3)	3.68 (2.67–5.05)	*p* < 0.001	20.8 (15.8–26.8)	0.82 (0.53–1.26)	0.367	30.0 (25.6–34.9)	2.80 (1.96–4.01)	*p* < 0.001
**Sex**									
Male	56.3 (52.1–60.4)	Ref		58.9 (52.1–65.4)	Ref		59.6 (54.5–64.4)	Ref	
Female	43.7 (39.6–47.8)	0.72 (0.59–0.89)	0.003	41.1 (34.5–47.9)	0.65 (0.48–0.89)	0.008	40.4 (35.6–45.5)	0.62 (0.49–0.79)	*p* < 0.001
**Number of under-five children**									
1	35.7 (31.7–39.8)	Ref		32.8 (26.6–39.7)	Ref		36.8 (31.9–41.9)	Ref	
2+	64.3 (60.1–68.2)	1.13 (0.91–1.41)	0.268	67.2 (60.3–73.4)	1.22 (0.87–1.69)	0.239	63.2 (58.1–68.1)	1.01 (0.78–1.31)	0.913
**Birth order**									
1	20.4 (17.2–24.1)	Ref		17.2 (12.5–23.1)	Ref		20.3 (16.5–24.8)	Ref	
2–4	48.7 (44.5–52.9)	1.17 (0.88–1.54)	0.269	41.4 (34.7–48.4)	1.14 (0.74–1.75)	0.561	43.7 (38.7–48.9)	1.03 (0.74–1.41)	0.873
4+	30.9 (27.1–34.9)	1.11 (0.82–1.51)	0.485	41.4 (34.7–48.4)	1.92 (1.22–2.99)	0.004	35.9 (31.1–41.0)	1.38 (0.97–1.94)	0.065
**Birth interval**									
< 24 months	20.0 (16.4–24.0)	Ref		17.2 (12.1–23.7)	Ref		17.9 (13.8–22.7)	Ref	
≥ 24 months	80.0 (75.9–83.5)	0.84 (0.62–1.14)	0.261	82.8 (76.2–87.9)	1.11 (0.71–1.74)	0.651	82.1 (77.2–86.1)	1.01 (0.71–1.44)	0.951
**Vitamin A in last 6 months**									
Yes	59.7 (55.4–63.8)	Ref		74.7 (68.1–80.4)	Ref		61.5 (56.3–66.4)	Ref	
No	40.3 (36.1–44.5)	1.43 (1.14–1.17)	0.002	25.3 (19.6–31.8)	0.60 (0.42–0.86)	0.006	38.5 (33.6–43.7)	1.21 (0.93–1.56)	0.146
**Currently breastfeeding**									
Yes	17.5 (14.5–20.9)	Ref		16.2 (11.6–21.9)	Ref		19.2 (15.5–23.6)	Ref	
No	82.5 (79.1–85.5)	1.28 (0.96–1.71)	0.088	83.8 (78.0–88.3)	1.06 (0.69–1.62)	0.780	80.8 (76.4–84.5)	1.53 (1.11–2.11)	0.010
**Minimum meal frequency**									
Yes	53.2 (49.1–57.3)	Ref		39.6 (33.1–46.4)	Ref		48.1 (43.1–53.2)	Ref	
No	46.8 (42.6–50.9)	0.64 (0.52–0.80)	*p* < 0.001	60.4 (53.5–66.8)	1.40 (1.02–1.93)	0.037	51.9 (46.7–56.9)	0.93 (0.73–1.19)	0.558
**Minimum dietary diversity**									
Yes	7.6 (5.6–10.2)	Ref		3.5 (1.7–7.2)	Ref		5.8 (3.8–8.8)	Ref	
No	92.4 (89.8–94.3)	1.13 (0.76–1.67)	0.542	96.5 (92.7–98.3)	2.75 (1.24–6.07)	0.012	94.1 (91.2–96.1)	1.57 (0.95–2.58)	0.076
**Maternal factors**									
**Age (years)**									
< 20	8.9 (6.8–11.6)	Ref		10.6 (7.0–15.7)	Ref		10.6 (7.8–14.2)	Ref	
20–34	75.6 (71.8–79.1)	0.91 (0.62–1.34)	0.654	70.7 (63.9–76.6)	0.72 (0.43–1.20)	0.209	71.0 (66.1–75.5)	0.76 (0.50–1.15)	0.198
35–49	15.4 (12.6–18.7)	0.84 (0.54–1.32)	0.449	18.7 (13.8–24.7)	0.98 (0.53–1.79)	0.942	18.4 (14.7–22.7)	0.97 (0.59–1.58)	0.910
**Educational level**									
No education	55.5 (51.3–59.7)	1.35 (1.08–1.68)	0.008	64.1 (57.2–70.5)	1.91 (1.37–2.65)	0.002	59.6 (54.4–64.6)	1.67 (1.29–2.15)	*p* < 0.001
Primary and above	44.4 (40.3–48.6)	Ref		35.8 (29.4–42.7)	Ref		40.4 (35.4–45.6)	Ref	
**ANC visit**									
Yes	71.8 (67.7–75.4)	Ref		60.2 (53.1–66.9)	Ref		67.5 (62.4–72.2)	Ref	
No	28.2 (24.5–32.2)	1.26 (0.98–1.62)	0.075	39.8 (33.1–46.9)	1.98 (1.42–2.78)	*p* < 0.001	32.5 (27.7–37.6)	1.58 (1.19–2.10)	0.001
**Place of delivery**									
Home	49.2 (45.0–53.5)	1.32 (1.05–1.66)	0.019	54.6 (47.5–61.4)	1.52 (1.09–2.12)	0.013	54.5 (49.3–59.6)	1.72 (1.31–2.24)	*p* < 0.001
Health facility	50.8 (46.5–54.9)	Ref		45.4 (38.5–52.4)	Ref		45.5 (40.3–50.7)	Ref	
**Household-level factors**									
**Household size**									
1–4	30.3 (26.5–34.3)	Ref		25.3 (19.7–31.7)	Ref		27.9 (23.4–32.7)	Ref	
5+	69.7 (65.7–73.4)	1.08 (0.85–1.36)	0.527	74.7 (68.2–80.3)	1.39 (0.97–1.98)	0.065	72.1 (67.3–76.5)	1.21 (0.93–1.59)	0.160
**Wealth index**									
Poorest	34.4 (30.5–38.5)	2.40 (1.69–3.41)	*p* < 0.001	50.5 (43.5–57.4)	3.09 (1.89–5.03)	*p* < 0.001	42.1 (37.0–47.2)	2.98 (1.99–4.47)	*p* < 0.001
Poorer	19.9 (16.7–23.5)	2.64 (1.81–3.86)	*p* < 0.001	16.2 (11.6–21.9)	1.80 (1.02–3.19)	0.043	19.7 (15.9–24.2)	2.55 (1.63–3.98)	*p* < 0.001
Middle	16.5 (13.6–19.9)	2.63 (1.78–3.88)	*p* < 0.001	8.1 (5.0–12.8)	1.02 (0.52–1.99)	0.945	12.2 (9.2–16.1)	1.76 (1.09–2.85)	0.021
Richer	15.2 (12.4–18.5)	2.41 (1.63–3.57)	*p* < 0.001	10.6 (7.0–15.7)	1.39 (0.75–2.58)	0.298	12.2 (9.2–16.0)	1.81 (1.13–2.92)	0.014
Richest	13.9 (11.2–17.1)	Ref		14.6 (10.4–20.3)	Ref		13.6 (10.4–17.6)	Ref	
**Toilet facility**									
Improved	14.8 (12.0–18.1)	Ref		16.3 (11.8–22.2)	Ref		15.8 (12.3–19.9)	Ref	
Unimproved	39.4 (35.4–43.7)	1.68 (1.21–2.32)	0.002	23.9 (18.5–30.4)	0.84 (0.51–1.38)	0.490	32.4 (27.7–37.4)	1.17 (0.80–1.71)	0.411
Open defecation	45.7 (41.5–50.0)	1.95 (1.41–2.71)	*p* < 0.001	59.7 (52.6–66.3)	2.09 (1.34–3.28)	0.001	51.8 (46.6–57.0)	1.98 (1.37–2.85)	*p* < 0.001
**Source of drinking water**									
Improved	14.8 (12.0–18.1)	Ref		16.3 (11.8–22.2)	Ref		15.8 (12.3–19.9)	Ref	
Unimproved	39.4 (35.4–43.7)	1.04 (0.76–1.44)	0.781	23.9 (18.5–30.5)	1.67 (1.10–2.53)	0.015	32.3 (27.8–37.4)	1.09 (0.78–1.54)	0.590
Surface water	45.7 (41.5–50.0)	1.15 (0.85–1.54)	0.360	59.7 (52.6–66.3)	0.86 (0.55–1.36)	0.524	51.8 (46.6–57.0)	1.57 (1.12–2.21)	0.010
**Community-level factors**									
**Residence**									
Urban	15.6 (12.8–18.9)	Ref		21.2 (16.0–27.5)	Ref		19.2 (15.4–23.6)	Ref	
Rural	84.4 (81.1–87.2)	2.31 (1.68–3.17)	*p* < 0.001	78.8 (72.5–83.9)	1.25 (0.82–1.93)	0.293	80.8 (76.4––84.5)	1.62 (1.13–2.32)	0.009
**Region**									
City administration	14.8 (12.1–18.1)	0.53 (0.38–0.75)	*p* < 0.001	9.5 (6.2–14.5)	0.48 (0.28–0.84)	0.009	12.2 (9.2–16.7)	0.47 (0.31–0.71)	*p* < 0.001
Pastoralist	19.5 (16.4–23.1)	0.79 (0.56–1.11)	0.181	35.8 (29.4–42.8)	2.27 (1.52–3.39)	*p* < 0.001	22.8 (18.7–27.5)	1.07 (0.74–1.56)	0.696
Agrarian	65.6 (61.4–69.5)	Ref		54.5 (47.5–61.4)	Ref		64.9 (59.8–69.7)	Ref	

### Determinants of stunting among children aged 0–23 months

The odds of being stunted were lower in female children compared with male children [AOR: 0.68, 95% CI (0.54–0.86)]. The odds of stunting were also less likely among children born of mothers aged 35–49 years old [AOR: 0.57, 95% CI (0.27, 0.97)] than children born of younger mothers. The likelihood of being stunted among children aged 12–17 months [AOR: 2.22, 95% CI (1.52, 3.23)] and 18–23 months old [AOR: 4.16, 95% CI (2.75, 6.27)] were 2.22 and 4.16 times higher compared with children in age grouped less than 6 months. Likewise, the probability of being stunted was higher in children from the poorest [AOR: 2.06, 95% CI (1.12, 3.79)], poorer [AOR: 2.25, 95% CI (1.27, 3.97)], middle wealth quintile [AOR: 2.08, 95% CI (1.19, 3.65)], and richer households [AOR: 2.02, 95% CI (1.19, 3.42)] compared to those children richest households (Table 3).

**Table 3:** Multivariable multilevel logistic regression analysis of factors associated with childhood stunting among children aged 0–23 months in Ethiopia, EMDHS 2019.

Characteristics	Null model (Model 0)	Individual level model (Model 1), AOR (95% CI)	Community level model (Model 2), AOR (95% CI)	Full model (Model 3), AOR (95% CI)
**Child related factors**				
**Sex**				
Male		Ref		Ref
Female		0.68 (0.54–0.86)*		0.68 (0.54–0.86)*
**Age (months)**				
< 6		Ref		Ref
6–11		1.36 (0.94–1.98)		1.36 (0.93–1.97)
12–17		2.23 (1.54–3.25)**		2.22 (1.52–3.23)**
18–23		4.25 (2.82–6.40)**		4.16 (2.75–6.27)**
**Number of under-five children**				
1		Ref		Ref
2+		1.19 (0.92–1.56)		1.22 (0.93–1.59)
**Vitamin A in last 6 months**				
Yes		Ref		Ref
No		1.14 (0.88–1.49)		1.13 (0.87–1.47)
**Currently breastfeeding**				
Yes		Ref		Ref
No		0.82 (0.58–1.17)		0.85 (0.59–1.21)
**Minimum meal frequency**				
Yes		Ref		Ref
No		0.89 (0.66–1.18)		0.88 (0.65–1.17)
**Minimum dietary diversity**				
Yes		Ref		Ref
No		1.29 (0.84–2.00)		1.30 (0.84–2.01)
**Maternal factors**				
**Age (years)**				
< 20		Ref		Ref
20–34		0.75 (0.49–1.15)		0.74 (0.48–1.14)
35–49		0.59 (0.35–1.01)		0.57 (0.27–0.97)*
**Educational level**				
No education		1.26 (0.95–1.65)		1.29 (0.98–1.70)
Primary and above		Ref		Ref
**ANC visit**				
Yes		0.95 (0.70–1.29)		0.93 (0.68–1.26)
No		Ref		Ref
**Place of delivery**				
Home		0.98 (0.73–1.31)		0.97 (0.72–1.30)
Health facility		Ref		Ref
**Household-level factors**				
**Household size**				
1–4		Ref		Ref
5+		0.95 (0.72–1.25)		0.94 (0.72–1.24)
**Wealth index**				
Poorest		2.32 (1.37–3.93)*		2.06 (1.12–3.79)*
Poorer		2.73 (1.68–4.42)**		2.25 (1.27–3.97)*
Middle		2.52 (1.57–4.03)**		2.08 (1.19–3.65)*
Richer		2.36 (1.49–3.73)**		2.02 (1.19–3.42)*
Richest		Ref		Ref
**Toilet facility**				
Improved		Ref		Ref
Unimproved		1.11 (0.76–1.63)		1.12 (0.73–1.73)
Open defecation		1.19 (0.78–1.82)		1.01 (0.68–1.51)
**Source of drinking water**				
Improved		Ref		Ref
Unimproved		1.06 (0.77–1.46)		0.82 (0.57–1.18)
Surface water		0.80 (0.56–1.15)		1.08 (0.78–1.48)
**Community-level factors**				
**Residence**				
Urban			Ref	Ref
Rural			2.09 (1.48–2.95)**	1.19 (0.75–1.89)
**Region**				
City administration			0.75 (0.52–1.09)	0.82 (0.54–1.24)
Pastoralist			0.81 (0.58–1.13)	0.73 (0.48–1.11)
Agrarian			Ref	Ref
**Random effect**				
Community variance (SE)	0.4200 (0.008)	0.3102 (0.0103)	0.3333 (0.0081)	0.3092 (0.0103)
ICC (%)	11.32	8.62	9.19	8.59
MOR	1.85	1.69	1.73	1.69
PCV (%)	Ref	26.14	20.64	26.38
**Model fit statistics**				
LL	− 1157.2118	− 990.348	− 1141.2625	− 988.614
DIC(-2Log-likelihood)	2,314.42	1,980.69	2,282.52	1,977.22
AIC	2318.42	2030.698	2292.525	2033.229
BIC	2329.64	2168.906	2320.582	2188.022

**p-value < 0.001; *p-value < 0.05; *SE* Standard Error; *ICC* Intra-class Correlation Coefficient; *MOR* Median Odds Ratio; *PCV* Proportional Change in Variance; *AIC* Akaike’s Information Criterion; *BIC* Bayesian information Criteria; *DIC* Deviance Information Criterion; *LL* Log-likelihood.

### Determinants of wasting among children aged 0–23 months

Being a female child [AOR: 0.70, 95% CI (0.51, 0.98)], a child residing in the rural area [AOR: 0.49, 95% CI (0.92, 0.92)], and a child living in city administrations [AOR: 0.49, 95% CI (0.26, 0.92) were associated with lower odds of being wasted (Table 4).

**Table 4:** Multivariable multilevel logistic regression analysis of factors associated with childhood wasting among children aged 0**–**23 months in Ethiopia, EMDHS 2019.

Characteristics	Null model (Model 0)	Individual level model (Model 1), AOR (95% CI)	Community level model (Model 2), AOR (95% CI)	Full model (Model 3), AOR (95% CI)
**Child related factors**				
**Sex**				
Male		Ref		Ref
Female		0.69 (0.49–0.96)*		0.70 (0.51–0.98)*
**Age (months)**				
< 6		Ref		Ref
6–11		0.83 (0.51–1.37)		0.81 (0.49–1.34)
12–17		1.07 (0.65–1.76)		1.03 (0.63–1.70)
18–23		1.28 (0.73–2.26)		1.30 (0.74–2.29)
**Number of under-five children**				
1		Ref		Ref
2+		0.88 (0.59–1.30)		0.88 (0.59–1.29)
**Vitamin A in last 6 months**				
Yes		Ref		Ref
No		0.77 (0.51–1.15)		0.79 (0.52–1.19)
**Currently breastfeeding**				
Yes		Ref		Ref
No		0.93 (0.56–1.57)		0.89 (0.53–1.52)
**Minimum meal frequency**				
Yes		Ref		Ref
No		1.17 (0.76–1.80)		1.16 (0.75–1.78)
**Minimum dietary diversity**				
Yes		Ref		Ref
No		2.26 (0.94–5.46)		2.21 (0.91–5.34)
**Maternal factors**				
**Age (years)**				
< 20		Ref		Ref
20–34		0.76 (0.43–1.37)		0.78 (0.44–1.40)
35–49		0.78 (0.38–1.59)		0.81 (0.40–1.66)
**Educational level**				
No education		1.32 (0.88–1.97)		1.32 (0.87–1.98)
Primary and above		Ref		Ref
**ANC visit**				
Yes		0.65 (0.43–0.99)		0.68 (0.45–1.04)
No		Ref		Ref
**Place of delivery**				
Home		0.81 (0.52–1.24)		0.83 (0.53–1.28)
Health facility		Ref		Ref
**Household-level factors**				
**Household size**				
1–4		Ref		Ref
5+		1.44 (0.95–2.20)		0.94 (0.72–1.24)
**Wealth index**				
Poorest		1.61 (0.77–3.36)		1.76 (0.74–4.18)
Poorer		1.41 (0.69–2.85)		1.79 (0.78–4.10)
Middle		0.95 (0.44–2.03)		1.14 (0.47–2.71)
Richer		1.25 (0.62–2.49)		1.48 (0.68–3.24)
Richest		Ref		Ref
**Toilet facility**				
Improved		Ref		Ref
Unimproved		0.66 (0.37–1.16)		0.68 (0.38–1.22)
Open defecation		1.26 (0.71–2.25)		1.29 (0.72–2.32)
**Source of drinking water**				
Improved		Ref		Ref
Unimproved		0.99 (0.62–1.58)		0.67 (0.41–1.09)
Surface water		0.67 (0.41–1.09)		0.96 (0.60–1.52)
**Community-level factors**				
**Residence**				
Urban			Ref	Ref
Rural			0.88 (0.56–1.38)	0.49 (0.92–0.92)*
**Region**				
City administration			0.45 (0.25–0.82)*	0.49 (0.26–0.92)*
Pastoralist			2.26 (1.51–3.38)**	1.43 (0.87–2.36)
Agrarian			Ref	Ref
**Random effect**				
Community variance (SE)	0.5722 (0.0178)	0.3587 (0.0263)	0.3999 (0.0192)	0.2989 (0.0279)
ICC (%)	14.81	9.83	10.84	8.33
MOR	2.05	1.76	1.82	1.68
PCV (%)	Ref	37.31	30.11	48.28
**Model fit statistics**				
LL	− 642.33	− 561.01	− 627.05	− 553.79
DIC(-2Log-likelihood)	1,284.66	1,122.02	1,254.1	1,107.58
AIC	1288.66	1172.003	1264.09	1163.583
BIC	1299.91	1310.479	1292.20	1318.676

**p-value < 0.001; *p-value < 0.05; *SE* Standard Error; *ICC* Intra-class Correlation Coefficient; *MOR* Median Odds Ratio; *PCV* Proportional Change in Variance; *AIC* Akaike’s Information Criterion; *BIC* Bayesian information Criteria; *DIC* Deviance Information Criterion; *LL* Log-likelihood.

### Determinants of underweight among children aged 0–23 months

The odds of being underweight were lower in female children compared with male children [AOR: 0.64, 95% CI (0.49, 0.83)]. The likelihood of being underweight was higher in older age groups: 6–11 months [AOR: 1.74, 95% CI (1.15, 2.63)], 12–17 months [AOR: 2.13, 95% CI (1.40, 3.24)], and 18–23 months [AOR: 4.08, 95% CI (2.58, 6.44)] compared with the ages group of less than 6 months. Also, a child from households from the poorest [AOR: 2.28, 95% CI (1.14, 4.54)] and poorer wealth quartiles [AOR: 2.42, 95% CI (1.26, 4.63)] were higher odds of being underweight. Moreover, children living in city administrations [AOR: 0.57, 95% CI (0.35, 0.93)] were significantly associated with lowers odds of being underweight (Table 5).

**Table 5:** Multivariable multilevel logistic regression analysis of factors associated with childhood underweight among children aged 0–23 months in Ethiopia, EMDHS 2019.

Characteristics	Null model (Model 0)	Individual level model (Model 1), AOR (95% CI)	Community level model (Model 2), AOR (95% CI)	Full model (Model 3), AOR (95% CI)
**Child related factors**				
**Sex**				
Male		Ref		Ref
Female		0.64 (0.49–0.83)*		0.64 (0.49–0.83)*
**Age (months)**				
< 6		Ref		Ref
6–11		1.78 (1.18–2.70)*		1.74 (1.15–2.63)*
12–17		2.19 (1.44–3.34)**		2.13 (1.40–3.24)**
18–23		4.19 (2.65–6.64)**		4.08 (2.58–6.44)**
**Number of under-five children**				
1		Ref		Ref
2+		0.86 (0.64–1.17)		0.89 (0.66–1.20)
**Vitamin A in last 6 months**				
Yes		Ref		Ref
No		1.10 (0.81–1.48)		1.09 (0.81–1.47)
**Currently breastfeeding**				
Yes		Ref		Ref
No		1.13 (0.76–1.67)		1.15 (0.78–1.71)
**Minimum meal frequency**				
Yes		Ref		Ref
No		1.40 (1.02–1.94)*		1.36 (0.99–1.88)
**Minimum dietary diversity**				
Yes		Ref		Ref
No		1.46 (0.85–2.52)		1.49 (0.87–2.55)
**Maternal factors**				
**Age (years)**				
< 20		Ref		Ref
20–34		0.71 (0.44–1.13)		0.70 (0.44–1.12)
35–49		0.75 (0.43–1.32)		0.72 (0.41–1.28)
**Educational level**				
No education		1.22 (0.89–1.66)		1.28 (0.94–1.76)
Primary and above		Ref		Ref
**ANC visit**				
Yes		0.91 (0.65–1.27)		0.90 (0.64–1.26)
No		Ref		Ref
**Place of delivery**				
Home		1.16 (0.83–1.62)		1.19 (0.85–1.67)
Health facility		Ref		Ref
**Household-level factors**				
**Household size**				
1–4		Ref		Ref
5+		1.18 (0.86–1.63)		1.16 (0.84–1.59)
**Wealth index**				
Poorest		1.95 (1.08–3.51)*		2.28 (1.14–4.54)*
Poorer		2.17 (1.25–3.77)*		2.42 (1.26–4.63)*
Middle		1.57 (0.89–2.74)		1.72 (0.89–3.32)
Richer		1.55 (0.91–2.68)		1.72 (0.93–3.18)
Richest		Ref		Ref
**Toilet facility**				
Improved		Ref		Ref
Unimproved		0.81 (0.52–1.26)		0.76 (0.48–1.20)
Open defecation		1.06 (0.66–1.70)		1.05 (0.65–1.68)
**Source of drinking water**				
Improved		Ref		Ref
Unimproved		0.94 (0.65–1.37)		0.98 (0.68–1.41)
Surface water		1.04 (0.70–1.54)		1.07 (0.73–1.57)
**Community-level factors**				
**Residence**				
Urban			Ref	Ref
Rural			1.26 (0.85–1.85)	0.60 (0.36–1.01)
**Region**				
City administration			0.53 (0.33–0.82)*	0.57 (0.35–0.93)*
Pastoralist			1.08 (0.74–1.57)	0.75 (0.48–1.16)
Agrarian			Ref	Ref
**Random effect**				
Community-level variance (SE)	0.5049 (0.0093)	0.3971 (0.0119)	0.4588 (0.0094)	0.3330 (0.0125)
ICC (%)	13.30	10.77	12.23	9.19
MOR	1.96	1.81	1.90	1.73
PCV (%)	Ref	21.35	9.11	34.04
**Model fit statistics**				
LL	− 935.70	− 818.23	− 927.47	− 813.99
DIC(-2Log-likelihood)	1,871.40	1,636.46	1,854.94	1,627.98
AIC	1875.41	1686.456	1864.937	1683.986
BIC	1886.66	1825.051	1893.067	1839.212

**p-value < 0.001; *p-value < 0.05; *SE* Standard Error; *ICC* Intra-class Correlation Coefficient; *MOR* Median Odds Ratio; *PCV* Proportional Change in Variance; *AIC* Akaike’s Information Criterion; *BIC* Bayesian information Criteria; *DIC* Deviance Information Criterion; *LL* Log-likelihood.

### Random effect analysis

The Intraclass Correlation Coefficient (ICC) in the null model for stunting, wasting and underweight was 11.32%, 14.81%, and 13.30%, respectively, which shows the variability of the conditions attributed to the clustering effect. The median odds ratio (MOR) value of the null models for stunting, wasting and underweight were 1.85, 2.05, and 1.96, respectively, which also indicates the presence of variation in the prevalence of stunting, wasting and underweight between clusters. The MOR measures the unexplained cluster heterogeneity (the variation between clusters) by comparing two individuals from two randomly chosen different clusters. In addition, the proportional change in variance (PCV) of the final models for stunting, wasting and underweight were 26.38%, 48.28% and 34.04%, respectively, indicating that the full model best explains the variability. The smallest values of Log-likelihood, AIC, and BIC were observed in model 3 and this implies that the full model for childhood stunting, wasting and underweight was a better explanatory model. As a result, interpretations and reports were made based on model 3 (full model) (Tables 3, 4 and 5).

## Discussion

Undernutrition is unacceptably high in Ethiopia, especially among young children^10,16,48,50^. When undernutrition occurs in the first 1000 days of life (0–23 months) it continues to the age of five and has long-term effects including physically, psychologically, culturally and socioeconomically^60^. In Ethiopia, evidence on the relative strengths of determinants of undernutrition in children aged 0 to 23 months is lacking at the national level, which is essential for understanding its underlying mechanisms. This study assessed the determinants of stunting (low height-for-age), wasting (low weight-for-height), and underweight (low weight-for-age) among children aged 0–23 months. The prevalence of stunting, wasting, and underweight was found to be 27.21%, 7.80%, and 16.44%, respectively. Multilevel multivariate analysis revealed that the most consistent significant risk factors for stunting, wasting, and underweight among children aged 0–23 months are: sex of the child, child’s age, maternal age, household-wealth status, and the type of place of residence.

The prevalence of stunting in the current study at 27.21% indicates that stunting continues to be major public health problem in the country. The observed prevalence of stunting in Ethiopia should be categorized as high as per international standards at a range between 20 to 30%. This level of stunting should be-a trigger-level as a basis of public health decisions^61^. This finding was consistent with studies carried out among infants between the ages of 0 and 23 months in Nigeria 29%^62^, Bangladesh 30.9%^63^, and Indonesia 28.4%^64^. However, although the prevalence of stunting in Ethiopia was higher than the international standards, was relatively lower than that of Indian infants aged 0–23 months (33%)^65^.

The observed prevalence of wasting and underweight was also in agreement with a study report from Indonesia^66^. However, higher prevalence of wasting (17%) was reported from the Dabat Health and Demographic Surveillance System (HDSS) site, northwest Ethiopia^39^, and Sahel region of Burkina Faso 25%^67^, among children 6–23 months. On the other hand, our finding was higher than the prevalence of wasting (2.0%) and underweight (2.1%) that was reported in China^68^. The current study findings were also higher than the prevalence of wasting (15.3%) and underweight (21.8%) reported in India^69^, and underweight prevalence reported in Bangladesh 24.9%^63^. The discrepancies in the results could be attributed to differences in sample size, study design, and other cultural, environmental and social determinants like dietary habits^70–72^.

In the current study, female children had reduced odds of being stunted, wasted and underweight than male counterparts. This finding concurred with previous observations in studies carried out in three East Africa countries (i.e. Rwanda, Uganda and Tanzania)^6^, and in similar studies conducted in Rwanda^73^, Kenya^74^, Nigeria^62^,Ghana^75^, Senegal^76^, and Indonesia^64^. Similar to the current study findings, all the above studies reported undernutrition to be higher in boys than in girls. In line with our findings, several studies in Ethiopia have also reported high odds of under-nutrition among male under-five children^27,41,47,77^, an indication that male children are more vulnerable to these conditions. Likewise, a study in Indonesia reported that the prevalence was higher in boys compare to girls (32.6 vs.14.2%, 25.9 vs 9.4% and 27.0 vs 18.0%), respectively for undernutrition, stunting and wasting^78^. Evidence suggests that this gender-based health disparity may be due to men being more vulnerable than women to various infections^79–81^.

In this study, an increase in the age of the child was positively associated with childhood stunting and being underweight, which is consistent with several other related studies conducted elsewhere^6,37,82–85^. The undernutrition attributed to the older can be explained by two major factors: 1) A lack of adequate and balanced food intake to meet the metabolic demand needed for childhood growth as they age; and 2) older child's frequent interactions with their surroundings, which may increase the risks of infections and exposure to childhood diseases, needing more nutrients for both growth and to fight infections^6,86^. For example, older children under five are vulnerable to diarrheal diseases, parasitic infections, and other acute illnesses, which potentiate the likelihood of stunting and underweight status. Furthermore, as the child grows and after the first six months, the breast milk becomes insufficient to meet nutritional needs, and if complementary foods are not introduced as needed, this change from exclusively breastfeeding to complementary family food can leave a child vulnerable to undernutrition. Therefore, in order to reduce childhood undernutrition, interventions aimed at improving child nutrition may need to focus on children that are starting complimentary feeding.

Our findings revealed that the odds of being stunted was also less likely among children born of middle age adults (35–49 years old) than children born of younger mothers. We hypothesis that this result could be explained by the fact that adult and well-mature mothers could have adequate knowledge about issues related to undermatron and may have significant experience towards infant and effective child feeding practices. Additionally, it is plausible to argue that this age group of mothers is possibly better educated than young Ethiopian girls (15–19 years old) and may be aware of their children's dietary demands and requirements. Among the well-known links between education and child health are that mothers' formal education directly provides an opportunity for them to understand health knowledge, improving their ability to identify illness and seek treatment for their children^87^. In support of this, several studies across various settings have shown that maternal education is one of the significant factors in providing protective effects against all childhood undernutrition indicators^31,42,47,88–90^.

In addition, the likelihood of having childhood growth retardation decreased as the wealth index increased. For example, children from richer families were less likely to be stunted or underweight. Several previous studies conducted in Ethiopia^47^, Tanzania^88^, Ghana^75^, Rwanda^89^, and Indonesia^64^ reported a positive association between higher wealth status and reduced odds of undernutrition. This relationship could reflect that higher wealth ensures adequate nutritious food supply and other required variables for effective growth in the households, while children from low-socioeconomic status families are less likely to have access to optimal nutrition, hence more likely to suffer from poor nutritional status.

According to the findings of this study, children who lived in rural areas had a decreased odds of being wasted. The notable difference in the rate of childhood wasting among urban and rural children could be explained satisfactorily by differences in the mothers' lifestyles from the Ethiopian context as follows: (i) rural-dwelling mothers spend much more time with their infants and breastfed regularly. (ii) The majority of rural mothers were both dependent on agriculture or raising livestock. With these backgrounds, it is likely that their children would have better access to animal dairy products such as milk, better supporting the rural children’s nutritional status. (iii) Mothers in rural areas currently receive home-visiting assistance and counseling from health extension workers. Furthermore, most urban mothers are employed and have limited time to care for their children, limiting regular breastfeeding, which in the absence of proper supplementary feeds can potentially lead to infection and undernutrition.

Furthermore, this study found that, compared to children living in agrarian regions, those born to mothers who resided in cities (such as Addis Ababa, the capital city) were less likely to be wasted and underweight. This pattern could relate to the fact that the mothers who live in cities have higher socioeconomic status, better housing conditions, and better access to adequate water and sanitation, all of which contribute to their children's health and nutritional quality.

There are several limitations to our analysis. Firstly, we used the DHS dataset, which is the cross-sectional data. As such, the analysis of a cross sectional study could not provide evidence of a causal relationship between outcome and independent variables. Secondly, data on personal and household practices were based on the mothers’ recall, which might have been subject to recall bias. Thirdly, the analysis did not include all determinants of childhood undernutrition, such as household water quality, maternal nutritional status, childhood-related illnesses and underlying disease conditions due to lack of data detailing these variables in the DHS. Fourthly, despite the use of a comprehensive set of variables in the analysis, the influence of residual confounding as a result of unmeasured covariates (such as childhood infections) was not addressed because childhood malnutrition has multifactorial determinants. Despite these limitations, the study's data was collected across the country, making it nationally representative. Furthermore, we applied a multilevel analysis to the model, which is more appropriate for cluster data.

## Conclusions

The undernutrition scourge remains a higher public health issue of significance among children aged 0–23 months in Ethiopia. Our analyses identified, female children were less likely to be predisposed to stunting, wasting, and underweight compared to the male counterparts. Children susceptibility to stunting and underweight was associated with age, with older aged children being more likely to be stunted and underweight compared to younger children. Children from the poorest household wealth index had higher odds of being stunted and underweight than children from the richest households. As a result, reducing undernutrition in early life in Ethiopia necessitates nutrition interventions tailored to at-risk populations (i.e. those who are about to begin complementary feeding (6–23 months), male children, and those living in poverty). Nutrition-sensitive conditional cash transfer interventions that target low-income households with the male children are needed for Ethiopia to meet the Sustainable Development Goals (SDGs) 1, 2 and 3 by 2030. Moreover, addressing the nutritional problem in the first 1000 days (0–23 months) ensures the best possible time frame for children in Ethiopia, with long-term benefits.

## Supplementary Information

Supplementary Information.

## Data Availability

The datasets analysed during the current study are available in the Measure DHS website https://dhsprogram.com after formal online registration and submission of the project title and detailed project description.

## Abbreviations

AOR: Adjusted odds ratio
ANC: Antenatal care visits
BMI: Body mass index
CI: Confidence interval
EDHS: Ethiopian Demographic and Health Surveys
SNNP: Southern Nations and Nationalities and People
WHO: World Health Organization
