# Supplementary material for: Determinants of undernutrition among young children in Ethiopia
Source: Sci Rep. 2022 Dec 5;12:20945. doi: 10.1038/s41598-022-25160-y (PMC9722653; doi:10.1038/s41598-022-25160-y)
Supplement: Supplementary file 1 — Supplementary Information. [file 41598_2022_25160_MOESM1_ESM.docx]

**Supplementary File 1: Bivariable multilevel logistic regression analysis of factors associated with childhood stunting, wasting, and underweight among children aged 0-23 months in Ethiopia, EMDHS 2019**

| **Characteristics** | **Overall Stunting** | | | | **Overall Wasting** | | | | **Overall Underweight** | | | |
| --- | --- | --- | --- | --- | --- | --- | --- | --- | --- | --- | --- | --- |
|  | **Stunted** | **Not stunted** | **OR (95%CI)** | **p-value** | **Wasted** | **Not wasted** | **OR (95%CI)** | **p-value** | **Underweight** | **Not underweight** | **OR (95%CI)** | **p-value** |
| **Child-related** |  |  |  |  |  |  |  |  |  |  |  |  |
| **Age (months)** |  |  |  |  |  |  |  |  |  |  |  |  |
| < 6 | 89 | 434 | Ref. |  | 48 | 471 | Ref. |  | 52 | 484 | Ref. |  |
| 6-11 | 132 | 361 | 1.46 (1.06-2.02) | 0.021 | 31 | 470 | 0.70 (0.46-1.08) | 0.111 | 86 | 413 | 1.41 (0.97-2.03) | 0.069 |
| 12-17 | 170 | 396 | 2.18 (1.60-2.97) | p<0.001 | 49 | 523 | 0.77 (0.51-1.16) | 0.223 | 119 | 450 | 1.68 (1.18-2.39) | 0.004 |
| 18-23 | 167 | 303 | 3.68 (2.67-5.05) | p<0.001 | 32 | 441 | 0.82 (0.53-1.26) | 0.367 | 83 | 389 | 2.80 (1.96-4.01) | p<0.001 |
| **Sex** |  |  |  |  |  |  |  |  |  |  |  |  |
| Male | 326 | 704 | Ref. |  | 103 | 934 | Ref. |  | 208 | 838 | Ref. |  |
| Female | 233 | 792 | 0.72 (0.59-0.89) | 0.003 | 58 | 972 | 0.65 (0.48-0.89) | 0.008 | 134 | 899 | 0.62 (0.49-0.79) | p<0.001 |
| **Number of under-five children** |  |  |  |  |  |  |  |  |  |  |  |  |
| 1 | 196 | 593 | Ref. |  | 46 | 745 | Ref. |  | 118 | 679 | Ref. |  |
| 2+ | 346 | 841 | 1.13 (0.91-1.41) | 0.268 | 110 | 1,072 | 1.22 (0.87-1.69) | 0.239 | 214 | 986 | 1.01 (0.78-1.31) | 0.913 |
| **Birth order** |  |  |  |  |  |  |  |  |  |  |  |  |
| 1 | 124 | 332 | Ref. |  | 14 | 444 | Ref. |  | 63 | 399 | Ref. |  |
| 2-4 | 247 | 668 | 1.17 (0.88-1.54) | 0.269 | 72 | 836 | 1.14 (0.74-1.75) | 0.561 | 146 | 778 | 1.03 (0.74-1.41) | 0.873 |
| 4+ | 171 | 434 | 1.11 (0.82-1.51) | 0.485 | 71 | 536 | 1.92 (1.22-2.99) | 0.004 | 123 | 488 | 1.38 (0.97-1.94) | 0.065 |
| **Birth interval** |  |  |  |  |  |  |  |  |  |  |  |  |
| < 24 months | 84 | 187 | Ref. |  | 25 | 249 | Ref. |  | 53 | 220 | Ref. |  |
| ≥ 24 months | 328 | 915 | 0.84 (0.62-1.14) | 0.261 | 118 | 1,117 | 1.11 (0.71-1.74) | 0.651 | 215 | 1,040 | 1.01 (0.71-1.44) | 0.951 |
| **Vitamin A in last 6 months** |  |  |  |  |  |  |  |  |  |  |  |  |
| Yes | 340 | 949 | Ref. |  | 122 | 1,164 | Ref. |  | 219 | 1,087 | Ref. |  |
| No | 194 | 462 | 1.43 (1.14-1.17) | 0.002 | 34 | 625 | 0.60 (0.42-0.86) | 0.006 | 107 | 552 | 1.21 (0.93-1.56) | 0.146 |
| **Currently breastfeeding** |  |  |  |  |  |  |  |  |  |  |  |  |
| Yes | 457 | 1,249 | Ref. |  | 130 | 1,571 | Ref. |  | 272 | 1,450 | Ref. |  |
| No | 84 | 185 | 1.28 (0.96-1.71) | 0.088 | 26 | 245 | 1.06 (0.69-1.62) | 0.780 | 60 | 215 | 1.53 (1.11-2.11) | 0.010 |
| **Minimum meal frequency** |  |  |  |  |  |  |  |  |  |  |  |  |
| Yes | 286 | 659 | Ref. |  | 51 | 907 | Ref. |  | 154 | 802 | Ref. |  |
| No | 273 | 836 | 0.64 (0.52-0.80) | p<0.001 | 110 | 999 | 1.40 (1.02-1.93) | 0.037 | 188 | 935 | 0.93 (0.73-1.19) | 0.558 |
| **Minimum dietary diversity** |  |  |  |  |  |  |  |  |  |  |  |  |
| Yes | 52 | 149 | Ref. |  | 7 | 195 | Ref. |  | 28 | 176 | Ref. |  |
| No | 489 | 1,285 | 1.13 (0.76-1.67) | 0.542 | 150 | 1,621 | 2.75 (1.24-6.07) | 0.012 | 304 | 1,489 | 1.57 (0.95-2.58) | 0.076 |
| **Maternal factors** |  |  |  |  |  |  |  |  |  |  |  |  |
| **Age (years)** |  |  |  |  |  |  |  |  |  |  |  |  |
| <20 | 54 | 97 | Ref. |  | 8 | 146 | Ref. |  | 40 | 118 | Ref. |  |
| 20-34 | 400 | 1,054 | 0.91 (0.62-1.34) | 0.654 | 111 | 1,337 | 0.72 (0.43-1.20) | 0.209 | 216 | 1,248 | 0.76 (0.50-1.15) | 0.198 |
| 35-49 | 87 | 282 | 0.84 (0.54-1.32) | 0.449 | 37 | 333 | 0.98 (0.53-1.79) | 0.942 | 76 | 299 | 0.97 (0.59-1.58) | 0.910 |
| **Educational level** |  |  |  |  |  |  |  |  |  |  |  |  |
| No education | 287 | 628 | 1.35 (1.08-1.68) | 0.008 | 93 | 823 | 1.91 (1.37-2.65) | 0.002 | 189 | 737 | 1.67 (1.29-2.15) | p<0.001 |
| Primary and above | 255 | 806 | Ref. |  | 63 | 994 | Ref. |  | 143 | 928 | Ref. |  |
| **ANC visit** |  |  |  |  |  |  |  |  |  |  |  |  |
| Yes | 387 | 1,052 | Ref. |  | 96 | 1,338 | Ref. |  | 221 | 1,238 | Ref. |  |
| No | 137 | 354 | 1.26 (0.98-1.62) | 0.075 | 53 | 439 | 1.98 (1.42-2.78) | p<0.001 | 100 | 392 | 1.58 (1.19-2.10) | 0.001 |
| **Place of delivery** |  |  |  |  |  |  |  |  |  |  |  |  |
| Home | 266 | 616 | 1.32 (1.05-1.66) | 0.019 | 90 | 789 | 1.52 (1.09-2.12) | 0.013 | 184 | 702 | 1.72 (1.31-2.24) | p<0.001 |
| Health facility | 274 | 794 | Ref. |  | 67 | 1,001 | Ref. |  | 148 | 937 | Ref. |  |
| **Household-level factors** |  |  |  |  |  |  |  |  |  |  |  |  |
| **Household size** |  |  |  |  |  |  |  |  |  |  |  |  |
| 1-4 | 168 | 503 | Ref. |  | 32 | 634 | Ref. |  | 103 | 569 | Ref. |  |
| 5+ | 374 | 931 | 1.08 (0.85-1.36) | 0.527 | 124 | 1,183 | 1.39 (0.97-1.98) | 0.065 | 229 | 1,096 | 1.21 (0.93-1.59) | 0.160 |
| **Wealth index** |  |  |  |  |  |  |  |  |  |  |  |  |
| Poorest | 121 | 301 | 2.40 (1.69-3.41) | p<0.001 | 50 | 378 | 3.09 (1.89-5.03) | p<0.001 | 95 | 333 | 2.98 (1.99-4.47) | p<0.001 |
| Poorer | 124 | 302 | 2.64 (1.81-3.86) | p<0.001 | 37 | 385 | 1.80 (1.02-3.19) | 0.043 | 76 | 355 | 2.55 (1.63-3.98) | p<0.001 |
| Middle | 125 | 244 | 2.63 (1.78-3.88) | p<0.001 | 16 | 353 | 1.02 (0.52-1.99) | 0.945 | 63 | 311 | 1.76 (1.09-2.85) | 0.021 |
| Richer | 99 | 241 | 2.41 (1.63-3.57) | p<0.001 | 29 | 304 | 1.39 (0.75-2.58) | 0.298 | 58 | 284 | 1.81 (1.13-2.92) | 0.014 |
| Richest | 71 | 345 | Ref. |  | 23 | 395 | Ref. |  | 40 | 381 | Ref. |  |
| **Toilet facility** |  |  |  |  |  |  |  |  |  |  |  |  |
| Improved | 63 | 262 | Ref. |  | 23 | 302 | Ref. |  | 53 | 276 | Ref. |  |
| Unimproved | 273 | 751 | 1.68 (1.21-2.32) | 0.002 | 69 | 955 | 0.84 (0.51-1.38) | 0.490 | 148 | 891 | 1.17 (0.80-1.71) | 0.411 |
| Open defecation | 193 | 398 | 1.95 (1.41-2.71) | p<0.001 | 65 | 527 | 2.09 (1.34-3.28) | 0.001 | 128 | 466 | 1.98 (1.37-2.85) | p<0.001 |
| **Source of drinking water** |  |  |  |  |  |  |  |  |  |  |  |  |
| Improved | 352 | 907 | Ref. |  | 108 | 1,145 | Ref. |  | 207 | 1,061 | Ref. |  |
| Unimproved | 114 | 278 | 1.04 (0.76-1.44) | 0.781 | 21 | 375 | 1.67 (1.10-2.53) | 0.015 | 59 | 339 | 1.09 (0.78-1.54) | 0.590 |
| Surface water | 69 | 907 | 1.15 (0.85-1.54) | 0.360 | 26 | 279 | 0.86 (0.55-1.36) | 0.524 | 63 | 249 | 1.57 (1.12-2.21) | 0.010 |
| **Community-level factors** |  |  |  |  |  |  |  |  |  |  |  |  |
| **Residence** |  |  |  |  |  |  |  |  |  |  |  |  |
| Urban | 106 | 426 | Ref. |  | 37 | 488 | Ref. |  | 83 | 453 | Ref. |  |
| Rural | 436 | 1,008 | 2.31 (1.68-3.17) | p<0.001 | 119 | 1,328 | 1.25 (0.82-1.93) | 0.293 | 249 | 1,212 | 1.62 (1.13-2.32) | 0.009 |
| **Region** |  |  |  |  |  |  |  |  |  |  |  |  |
| City administration | 9 | 63 | 0.53 (0.38-0.75) | p<0.001 | 2 | 71 | 0.48 (0.28-0.84) | 0.009 | 4 | 73 | 0.47 (0.31-0.71) | p<0.001 |
| Pastoralist | 29 | 120 | 0.79 (0.56-1.11) | 0.181 | 25 | 125 | 2.27 (1.52-3.39) | p<0.001 | 28 | 123 | 1.07 (0.74-1.56) | 0.696 |
| Agrarian | 503 | 1,250 | Ref. |  | 130 | 1,620 | Ref. |  | 301 | 1,469 | Ref. |  |
